# Mixed Th1 and Th2 *Mycobacterium tuberculosis*-specific CD4 T cell responses in patients with active pulmonary tuberculosis from Tanzania

**DOI:** 10.1371/journal.pntd.0005817

**Published:** 2017-07-31

**Authors:** Patrizia Amelio, Damien Portevin, Klaus Reither, Francis Mhimbira, Maxmillian Mpina, Anneth Tumbo, Beatrice Nickel, Hanspeter Marti, Stefanie Knopp, Song Ding, Adam Penn-Nicholson, Fatoumatta Darboe, Khalid Ohmiti, Thomas J. Scriba, Giuseppe Pantaleo, Claudia Daubenberger, Matthieu Perreau

**Affiliations:** 1 Service of Immunology and Allergy, Lausanne University Hospital, University of Lausanne, Lausanne, Switzerland; 2 Swiss Tropical and Public Health Institute, Basel, Switzerland; 3 University of Basel, Basel, Switzerland; 4 Ifakara Health Institute, Bagamoyo, Tanzania; 5 EuroVacc Foundation, Lausanne, Switzerland; 6 South African Tuberculosis Vaccine Initiative, Institute of Infectious Disease and Molecular Medicine, Division of Immunology, Department of Pathology, University of Cape Town, South Africa; 7 SVRI, Lausanne, Switzerland; NIH-TRC-ICER, INDIA

## Abstract

*Mycobacterium tuberculosis* (*Mtb*) and helminth infections elicit antagonistic immune effector functions and are co-endemic in several regions of the world. We therefore hypothesized that helminth infection may influence *Mtb*-specific T-cell immune responses. We evaluated the cytokine profile of *Mtb*-specific T cells in 72 individuals with pulmonary TB disease recruited from two Sub-Saharan regions with high and moderate helminth burden *i*.*e*. 55 from Tanzania (TZ) and 17 from South Africa (SA), respectively. We showed that *Mtb*-specific CD4 T-cell functional profile of TB patients from Tanzania are primarily composed of polyfunctional Th1 and Th2 cells, associated with increased expression of Gata-3 and reduced expression of T-bet in memory CD4 T cells. In contrast, the cytokine profile of *Mtb*-specific CD4 T cells of TB patients from SA was dominated by single IFN-γ and dual IFN-γ/TNF-α and associated with TB-induced systemic inflammation and elevated serum levels of type I IFNs. Of note, the proportion of patients with *Mtb*-specific CD8 T cells was significantly reduced in *Mtb*/helminth co-infected patients from TZ. It is likely that the underlying helminth infection and possibly genetic and other unknown environmental factors may have caused the induction of mixed Th1/Th2 *Mtb*-specific CD4 T cell responses in patients from TZ. Taken together, these results indicate that the generation of *Mtb*-specific CD4 and CD8 T cell responses may be substantially influenced by environmental factors *in vivo*. These observations may have major impact in the identification of immune biomarkers of disease status and correlates of protection.

## Author summary

*Mycobacterium tuberculosis* (*Mtb*) and helminth infections are co-endemic in several regions of the world and their immune responses may be mutually antagonistic. We therefore hypothesized that helminth infection would impact and potentially shape *Mtb*-specific T-cell responses and systemic inflammation in patients suffering from active pulmonary tuberculosis (TB) enrolled from two helminth endemic regions *i*.*e*. Tanzania (TZ) and South Africa (SA). In this study, we demonstrate for the first time that TB patients from SA and TZ harbor distinct immune responses to *Mtb* antigens. Indeed, we showed that *Mtb*-specific CD4 T-cell responses of TB patients from TZ were composed by a mixed T helper type 1 (Th1) and Th2 responses. In contrast, the cytokine profile of *Mtb*-specific CD4 T cells of TB patients from SA was dominated by Th1 cells and associated with TB-induced systemic inflammation and elevated serum levels of type I IFN. Taken together, these data indicate that *Mtb*-specific T-cell responses are diverse in human populations and can be strongly influenced by host and pathogen genetic background, co-infections and yet unknown environmental factors. Identification of correlates of risk and protection from TB disease will help in the rational development of protective T-cell based vaccines against TB, early monitoring TB treatment outcomes and focused follow up of high risk populations.

## Introduction

Helminth infections are endemic in many African countries with different prevalence depending on the geographic region helminth species and age of population [1]. Soil transmitted helminth infections are among the most common infections, transmitted *via* soil contaminated by eggs excreted from human faeces [2]. Of note, helminth infections are co-endemic in many geographic areas endemic for *Mycobacterium tuberculosis* (*Mtb*), HIV-1 and *Plasmodium falciparum*. Therefore, co-infections of helminths with *Mtb*, HIV-1 and/or *Plasmodium falciparum* occur in a large proportion of the subjects [3].

*Mycobacterium tuberculosis* (*Mtb*) is a facultative intracellular organism, obligate aerobe, infecting primarily lungs *via* the aerogenic route [4]. It has been recently estimated that 1.7 billion people are infected with *Mtb* among which 5–15% will develop tuberculosis disease (TB) [5]. To date, the only vaccine available to prevent TB disease consist of an attenuate strain of *M*. *bovis*, the Bacillus of Calmette et Guérin (BCG). While BCG immunization protects from life-threatening disseminated forms of TB disease in children, its efficacy in adults is inconsistent [6]. The protective components of *Mtb*-specific immunity are partially defined. Several studies have underscored the essential role of IL-12/IFN-γ axis in the protection against *Mtb* infection [7–10]. In addition, an efficient CD4 T-cell response probably associated with type 1 cytokine secretion is associated with the control of *Mtb* infection, since a severe reduction in the CD4 T cell number during HIV infection or the suppression of their function following anti-TNF-α therapy are associated with increased risk of TB reactivation [11, 12].

The current paradigm of human cellular immunity indicates that functionally-distinct CD4 T-cell populations are specifically involved against a variety of pathogens depending on their size and their intra- or extra-cellular localization. In this model, type 1 helper CD4 T cells (Th1 cells) intervene against viruses and intracellular pathogens, Th2 cells against parasites such as worms and Th17 cells against extracellular pathogens [13, 14] including bacteria and fungi [14, 15]. Consistent with this paradigm, the protective *Mtb*-specific T-cell response is usually ascribed to typical Th1 response with CD4 T cells producing cytokines such as IFN-γ or TNF-α that contribute to the recruitment of monocytes and granulocytes and activate the anti-microbial activity of macrophages [16, 17]. By contrast, helminth infections induce IL-4/IL-5 and IL-13 producing CD4 T cells and regulatory T cells [18–21] associated with the alternative activation of macrophages for repair of tissues injured upon migration of worms across different body compartments [1]. With regard to the generation of the functionally distinct T helper CD4 T cell populations, the pioneering studies from Romagnani and others [22, 23], clearly demonstrated the critical importance of the cytokine environment, rather than the specific antigen in the development of distinct T helper antigen-specific CD4 T cells [24, 25]. In this regard, the presence of an IL-4 cytokine background favored the development of Th2 specific to pathogens that usually induce the classical Th1 responses [23, 26].

Multiple studies performed in *Mtb*/helminth co-infected individuals have focused on the impact of helminth infection on 1) TB diagnosis [27–35], 2) reactivation of TB from latently infected individuals (LTBI) [36, 37] and 3) BCG vaccine immunogenicity [38–40]. In addition, it was recently shown that helminth infection may interfere and/or influence innate [41], cellular [42–44] and humoral [45] immune responses to *Mtb*. However limited information is available on the cytokine profile of *Mtb*-specific T-cell immune responses in subjects with *Mtb*/helminth co-infection.

In the present study, we investigated the cytokine profile of *Mtb*-specific immune response in patients with clinically active TB from two countries *i*.*e*. Tanzania (TZ) and South Africa (SA) in the presence or absence of active helminth infections. We provide evidence that the functional profile of *Mtb*-specific CD4 T cells of TB patients from TZ was characterized by a mixed Th1/Th2 cytokine profile, while that from SA was associated with a typical single IFN-γ and dual IFN-γ/TNF-α Th1 profile.

These results demonstrate that distinct functional profiles of CD4 T-cell responses can be directed against the same pathogen, *i*.*e*. *Mtb*, in human populations from different geographic areas. *Ad hoc* designed studies will be needed to define the factors driving the distinct functional profiles as well as to determine whether the distinct functional profiles are associated with variation of the TB pathology and/or response to drug therapy.

## Results

The aims of the present study were i) to delineate *Mtb*-specific T-cell responses in patients with pulmonary tuberculosis (TB) and helminth co-infection from two Sub-Saharan countries, *e*.*g*. Tanzania (TZ) and South Africa (SA) and ii) to determine the influence of ongoing or past helminth infections on *Mtb*-specific T-cells responses. Therefore, we analyzed the cytokine profile and cell lineage T cell transcription factor expression in *Mtb*-specific CD4 T cells and serum cytokine levels in 72 individuals (Table 1). The patients were screened for active helminth infections and/or previous exposure to helminths and the cohort stratified into three groups: 1) active TB patients from SA with no sign of active helminth infections, 2) active TB patients from TZ with no sign of active helminth infections and 3) active TB patients co-infected with helminths from TZ (see flow chart, S1 Fig).

**Table 1:** Demographic and clinical data.

ID	Origin	Number	Mean Age	Gender	Helminth infection	BCG vaccination status
**TB SA**	South Africa	17	36	3F/12M	11% past exposure	17/17 (100%)
**TB TZ**	Tanzania	25	29	9F/16M	28% past exposure	25/25 (100%)
***Mtb*/helminthTZ**	Tanzania	30	30	6F/24M	Ongoing	30/30 (100%)

### *Mtb*-specific CD4 T cells from TB patients from TZ have mixed Th1/Th2 cytokine profile

**Fig 1:**
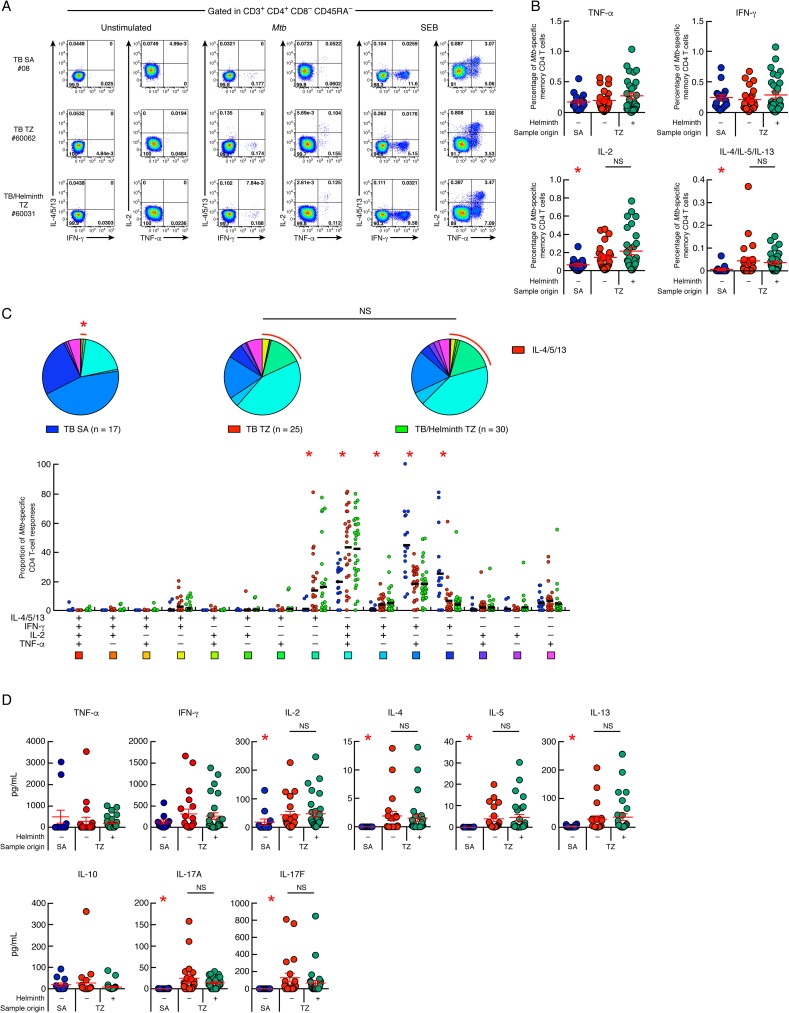
*Mtb*-specific CD4 T cells from TB patients from TZ have mixed Th1/Th2 cytokine profile. (**A**) Representative flow cytometry profile of *Mtb*-specific CD4 T cells producing IFN-γ, IL-4/5/13, TNF-α and/or IL-2 of one TB patient from SA (#08), one TB patients from TZ (#60062) and one *Mtb*/helminth co-infected patient from TZ (#60031). Cytokine profiles of CD4 T cells stimulated with SEB (positive control) or left unstimulated (negative control) are also shown. (**B**) Percentage of *Mtb*-specific CD4 T cells producing TNF-α, IFN-γ, IL-2 or IL-4/5/13 of TB patients from SA (n = 17), TB patients from TZ (n = 25) and *Mtb*/helminth co-infected patients from TZ (n = 30). (**C**) Proportion of *Mtb*-specific CD4 T-cell responses producing IFN-γ, IL-4/5/13, TNF-α and/or IL-2 of TB patients from SA (n = 17), TB patients from TZ (n = 25) and *Mtb*/helminth co-infected patients from TZ (n = 30). All the possible combinations of the responses are shown on the x axis and the percentage of the functionally distinct cell populations within the *Mtb*-specific CD4 T-cell populations are shown on the y axis. Responses are grouped and color-coded on the basis of the number of functions. The pie chart summarizes the data, and each slice corresponds to the fraction of *Mtb*-specific CD4 T cell response with a given number of functions within the responding CD4 T-cell population. Bars correspond to the fractions of different functionally distinct CD4 T-cell populations within the total CD4 T cells. Red arcs correspond to IL-4/5/13-producing CD4 T-cell populations. (**D**) Levels of IFN-γ, TNF-α, IL-10, IL-2, IL-4, IL-5, IL-13, IL-17A and IL-17F produced in *Mtb*-stimulated culture supernatants of TB patients from SA (n = 12), TB patients from TZ (n = 21) and *Mtb*/helminth co-infected patients from TZ (n = 29) assessed by luminex assay. TB patients were color coded (**B** and **D**); TB patients from SA, blue; TB patients from TZ, red and *Mtb*/helminth co-infected patients, green. Red stars indicate statistical significance. Statistical significance (* = *P*<0.05) was calculated using one way Anova Kruskal-Wallis test followed by a Mann-Whitney test (**B** and **D**). Statistical analyses of the global cytokine profiles (pie charts, **C**) were performed by partial permutation tests using the SPICE software as described [97].

The functional profiles of *Mtb*-specific CD4 T-cell responses were assessed by intracellular cytokine staining (ICS) according to the gating strategy shown in S2 Fig. In particular, the ability of *Mtb*-specific CD4 T cells to produce IFN-γ, TNF-α, IL-2, IL-4, IL-5 and/or IL-13 in response to ESAT-6 and CFP-10 peptide pools stimulation was assessed by multi-parametric flow cytometry in 25 TB patients and 30 *Mtb*/helminth co-infected patients from TZ and compared to 17 TB patients from SA. Of note, Th2 cytokines *i*.*e*. IL-4, IL-5 and IL-13 were all assessed in the same flow cytometry fluorescence channel, which allowed the assessment of total Th2 cytokine production but prevented direct identification of individual IL-4, IL-5 or IL-13 *Mtb*-specific CD4 T-cell responses. Cytokine profiles of *Mtb*-specific CD4 T cells from three representative TB patients from SA (#08) and TZ (TB (#60062) and *Mtb*/helminth co-infected patient (#60031) are shown in Fig 1A. We first compared the frequencies of cytokine-producing *Mtb*-specific memory CD4 T cells from TB patients from SA *versus* TB patients from TZ (Fig 1B). The cumulative data showed a significantly higher IL-2^+^ and IL-4/IL-5/IL-13^+^ *Mtb*-specific memory CD4 T-cell frequencies in TB patients from TZ compared with TB patients from SA (*P*<0.05), while the frequencies of IFN-γ and TNF-α producing *Mtb*-specific memory CD4 T cells were not significantly different between TB patients from TZ and from SA (*P*>0.05; Fig 1B). Interestingly, no significant differences were observed for Th1 and Th2 cytokine producing CD4 T cells between TB patients from TZ with and without ongoing helminth co-infections (*P*>0.05) (Fig 1B).

We next analyzed the cytokine profile of *Mtb*-specific memory CD4 T cells of TB patients from SA and TZ (Fig 1C, pie charts). The cytokine profile of *Mtb*-specific memory CD4 T cells of TB patients from SA was significantly different from of TB patients from TZ (*P*<0.05; Fig 1C, pie charts). Again, no significant differences were observed between TB patients and *Mtb*/helminth co-infected patients from TZ (*P*>0.05) (Fig 1C, pie charts). In depth analysis showed that *Mtb*-specific CD4 T-cell responses of TB patients from TZ were significantly enriched in polyfunctional IFN-γ^+^IL-2^+^TNF-α^+^IL-4/5/13^-^ CD4 T cells (triple IFN-γ/IL-2/TNF-α *Mtb*-specific CD4 T cells) and in IFN-γ^-^IL-2^-^TNF-α^-^IL-4/5/13^+^ CD4 T-cell populations (single IL-4/5/13 *Mtb*-specific CD4 T cells) as compared to TB patients from SA (43–42% *versus* 20% for triple IFN-γ/IL-2/TNF-α and 14–16% *versus* 1% for single IL-4/5/13; *P*<0.05) (Fig 1C). In contrast, *Mtb*-specific CD4 T-cell responses of TB patients from SA were significantly enriched in IFN-γ^+^IL-2^-^TNF-α^+^IL-4/5/13^-^ (dual IFN-γ/TNF-α *Mtb*-specific CD4 T cells) and in IFN-γ^+^IL-2^-^TNF-α^-^IL-4/5/13^-^ CD4 T-cell populations (single IFN-γ *Mtb*-specific CD4 T cells) as compared to TB patients from TZ (about 45% *versus* 20–18% for dual IFN-γ/TNF-α and 25% *versus* 7–4% for single IFN-γ^+^; *P*<0.05) (Fig 1C). No significant differences were observed between the *Mtb*-specific CD4 T-cell cytokine profile of TB patients and *Mtb*/helminth co-infected patients from TZ (*P*>0.05) (Fig 1C). Of note, *Schistosoma mansoni*-specific CD4 T-cell responses were evaluated on *Mtb*/helminth co-infected individuals from TZ using *S*. *mansoni* soluble egg antigens (SEA) by polychromatic flow cytometry (n = 7). The results obtained showed that SEA-specific CD4 T-cell responses were dominated by single TNF-α and single IL-4/IL-5/IL-13-producing CD4 T cells, while polyfunctional IFN-γ^+^IL-2^+^TNF-α^+^ CD4 T cells represented less than 5% of total SEA-specific CD4 T-cell responses confirming previous observations [46, 47] (S3 Fig).

To better estimate the influence of 1) different helminth species, 2) infection caused by more than one helminth (polyparasitism), 3) differences in helminth lung migration capacity and 4) past helminth exposure on the generation of Th2 *Mtb*-specific CD4 T-cells, the proportion of IL-4/5/13-producing *Mtb*-specific CD4 T cells was compared between TB patients from TZ with helminth infection caused by one or more helminth species (S4A Fig), or between TB patients from TZ coinfected with helminth species exhibiting (hookworms and *Strongyloides stercoralis*) or not (*S*. *mansoni*, *Schistosoma haematobium* and *Wuchereria bancrofti*) lung migration capacity (S4B Fig). Similarly, the proportion of IL-4/5/13-producing *Mtb*-specific CD4 T cells was compared between TB patients from SA or TZ with evidence of past exposure to helminths *versus* TB patients with no sign of ongoing or past exposure to helminths (S4C and S4D Fig). The cumulative data showed that the proportion of single IL-4/5/13-producing *Mtb*-specific CD4 T cells from active TB cases was not influenced by 1) polyparasitism (including *W*. *bancrofti*, hookworm, *S*. *mansoni*, *S*. *haematobium* or *S*. *stercoralis*) (*P*>0.05) (S4A Fig), 2) by helminth infections caused by helminth species exhibiting lung migration capacity, or 3) by past exposure to helminths (S4B and S4C Fig).

To further characterize the cytokine profile of *Mtb*-specific T-cells, multiplex bead array analyses (luminex) were performed on supernatants of ESAT-6/CFP-10 peptide pool (*Mtb*)-stimulated cell cultures. The cumulative data showed that *Mtb*-stimulated cell culture supernatants of TB patients from TZ secreted similar levels of IFN-γ, TNF-α and IL-10 (*P*>0.05), but significantly higher levels of IL-2, IL-4, IL-5, IL-13, IL-17A and IL-17F than *Mtb*-stimulated cell culture supernatants of TB patients from SA (*P*<0.05; Fig 1D). However, no significant differences were observed between TB patients and *Mtb*/helminth co-infected patients from TZ (*P*>0.05; Fig 1D), confirming our flow cytometry analyses. In addition, the levels of Th2 cytokines secreted in *Mtb*-stimulated cell culture supernatants of *Mtb*/helminth patients from TZ was not influenced by individual species of helminth infections, by polyparasitism (*P*>0.05) (S4E Fig), by helminth species exhibiting lung migration capacity (S4F Fig), or by past exposure to helminths (S4G and S4H Fig).

Taken together, our data indicate that a high proportion of *Mtb*-specific CD4 T cells from Tanzanian TB patients have a mixed Th1/Th2 cytokine profile which is observed either in patients with active helminth infection or in a large proportion of patients with positive helminth serology. In contrast, *Mtb*-specific CD4 T cells from South African TB cases have a classical, previously described Th1 cytokine profile.

### Memory CD4 T cells of TB patients from TZ harbor increased Gata-3 and reduced T-bet expression

We then determined whether the development of *Mtb*-specific Th2 CD4 T cells was associated with changes in the expression of Th1 and Th2 cell lineage transcription factors, T-bet and Gata-3 respectively [48, 49].

**Fig 2:**
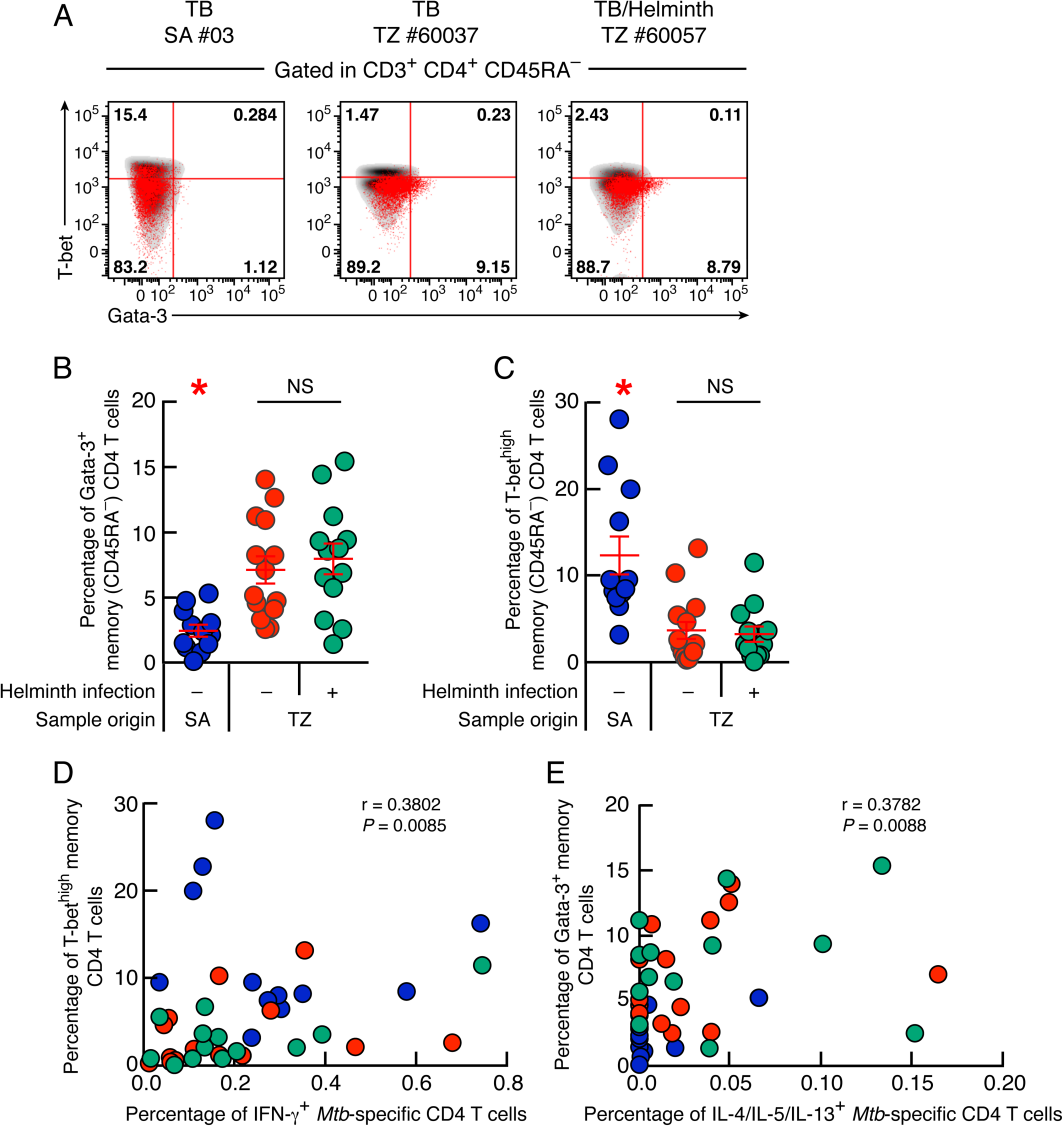
Memory CD4 T cells of TB patients from TZ harbor increased Gata-3 and reduced T-bet expression. (**A**) Representative flow cytometry profile of memory (CD45RA^-^) CD4 T cells (red dots) isolated from one TB patient from SA (#03), one TB patient (#60037) and one *Mtb*/helminth co-infected patient (#60057) from TZ expressing Gata-3 and/or T-bet. Gates were set using naïve (CD45RA^+^) CD4 T cells and CD8 T cells from each individual. Gata-3 and T-bet expression of individual CD8 T-cell are also shown (black dots). Percentage of memory (CD45RA^-^) CD4 T cells isolated from TB patients from SA (n = 12), TB patients (n = 14) and *Mtb*/helminth co-infected patients (n = 13) from TZ expressing Gata-3 (**B**) or T-bet^high^ (**C**). (**D**) Correlation between the percentage of IFN-γ-producing *Mtb*-specific CD4 T cells and the percentage of memory CD4 T cells expressing T-bet^high^ of TB patients from SA (n = 12), TB (n = 14) and *Mtb*/helminth co-infected patients from TZ (n = 13). (**E**) Correlation between the percentage of IL-4/5/13-producing *Mtb*-specific CD4 T cells and the percentage of memory CD4 T cells expressing Gata-3 of TB patients from SA (n = 12), TB (n = 14) and *Mtb*/helminth co-infected patients from TZ (n = 13). TB patients were color coded (**B**-**E**); TB patients from SA, blue; TB patients from TZ, red and *Mtb*/helminth co-infected patients, green. Red stars indicate statistical significance. Statistical significance (* = *P*<0.05) was calculated using one way Anova Kruskal-Wallis test followed by a Mann-Whitney test (**B** and **C**) or Spearman rank test for correlation (**D** and **E**).

Representative examples and cumulative data showed that the percentage of memory CD4 T cells expressing Gata-3 was significantly increased in TB and *Mtb*/helminth co-infected patients from TZ (7% and 7.9%, respectively) compared with TB patients from SA (2.4%; *P*<0.05) (Fig 2A and 2B). In contrast, the percentage of memory CD4 T cells expressing high levels of T-bet (T-bet^high^) was significantly lower in TB and *Mtb*/helminth patients from TZ (3.6% and 3.2%, respectively) as compared to TB patients from SA (12.3%; *P*<0.05) (Fig 2A and 2C). However, the frequencies of memory CD4 T cells expressing Gata-3 or T-bet^high^ did not differ between TB patients and *Mtb*/helminth co-infected patients from TZ (*P*>0.05) (Fig 2B and 2C). In addition, the frequency of memory CD4 T cells expressing Gata-3 of *Mtb*/helminth co-infected patients from TZ was not influenced by the helminth species and by polyparasitism (*P*>0.05) (S4I Fig), by helminth species exhibiting lung migration capacity (S4J Fig), or by past exposure to helminths (S4K and S4L Fig). Interestingly, the percentage of T-bet^high^ memory CD4 T cells negatively correlated with the percentage of memory CD4 T cells expressing Gata-3 (r = -0.6745; *P*<0.0001) (S5 Fig) thus supporting previous observations [50].

We then determined whether the expression of T-bet or Gata-3 by memory CD4 T cells was associated with *Mtb*-specific CD4 T-cell cytokine profile of TB patients and *Mtb*/helminth co-infected patients from SA or TZ. To address this issue, we plotted the percentage of *Mtb*-specific CD4 T cells producing IFN-γ or IL-4/5/13 against the percentage memory CD4 T cells expressing T-bet^high^ or Gata-3 from the same patients (Fig 2D and 2E). The cumulative data showed that the percentage of IFN-γ-producing *Mtb*-specific CD4 T cells directly correlated with the percentage of T-bet^high^ memory CD4 T cells (r = 0.3802, *P* = 0.0085) (Fig 2D) and the percentage of IL-4/5/13-producing *Mtb*-specific CD4 T cells directly correlated with the percentage of memory CD4 T cells expressing Gata-3 (r = 0.3782, *P*<0.0088) (Fig 2E).

Taken together, these data indicate that TB patients from TZ have a mixed Th1/Th2 cytokine profile associated with increased Gata-3 and reduced T-bet^high^ expression.

### Reduced proportion of patients with detectable *Mtb*-specific CD8 T cells in *Mtb*/helminth co-infected patients

**Fig 3:**
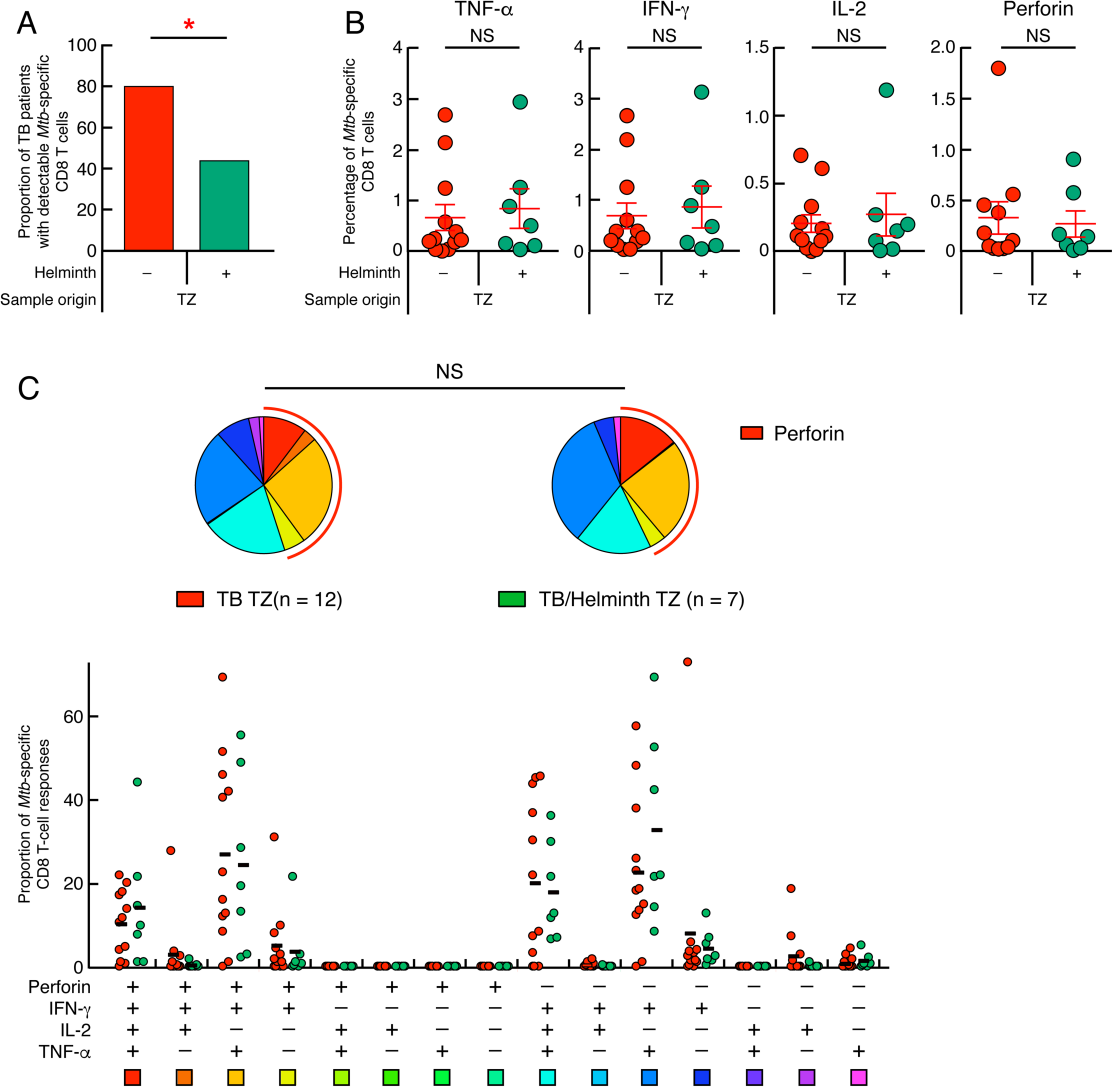
Reduced proportion of patients with detectable *Mtb*-specific CD8 T cells in *Mtb*/helminth co-infected patients. (**A**) Proportion of TB patients with detectable *Mtb*-specific CD8 T cells of TB and *Mtb*/helminth patients from TZ (n = 12 and 7, respectively). (**B**) Percentage of *Mtb*-specific CD8 T-cell responses producing TNF-α, IFN-γ, IL-2 and/or perforin of TB patients (n = 12) and *Mtb*/helminth patients (n = 7) from TZ. TB patients were color coded; TB patients from TZ, red and *Mtb*/helminth co-infected patients, green. (**C**) Functional profile of *Mtb*-specific CD8 T cells of TB patients and *Mtb*/helminth co-infected patients from TZ. Red and green circles represent the proportion of *Mtb*-specific CD8 T cells producing TNF-α, IFN-γ, IL-2 and/or perforin of TB and *Mtb*/helminth patients from TZ, respectively (**C**). Red arcs identify perforin producing cell populations (**C**). Red stars indicate statistical significance (* = *P*<0.05). Statistical significance (*P*<0.05) was calculated using Chi square test (**A**) and Mann-Whitney test (**B**). Statistical analyses of the global cytokine profiles (pie charts, **C**) were performed by partial permutation tests using the SPICE software as described [97].

Ongoing helminth infections has been shown to interfere with CD8 T cells responses targeting viruses in mouse models [51, 52]. Our group and others has recently shown that *Mtb*-specific CD8 T cells were more frequently detected in patients with TB disease as compared to those with latent *Mtb* infection [53]. Based on this observation, a recent diagnostic test *i*.*e*. QuantiFERON TB PLUS proposes optimized CD8-TB-specific-peptides stimulation [54, 55]. This prompted us to investigate whether ongoing helminth infections would influence the proportion of TB patients with detectable *Mtb*-specific CD8 T cells. To address this issue, the ability of *Mtb*-specific CD8 T cells to produce IFN-γ, TNF-α, IL-2 and perforin was assessed in 16 TB patients and 23 *Mtb*/helminth co-infected patients from TZ by flow cytometry. As shown in Fig 3A, the proportion of subjects with detectable *Mtb*-specific CD8 T cells was significantly reduced in *Mtb*/helminth co-infected patients from TZ as compared to TB patients from TZ (43.7% *versus* 80%, respectively; *P*<0.05; Fig 3A). The frequency of cytokine- and perforin-producing *Mtb*-specific CD8 T cells and the functional profile of *Mtb*-specific CD8 T-cell responses did not differ significantly between TB and *Mtb*/helminth co-infected patients from TZ with detectable *Mtb*-specific CD8 T cells (Fig 3B) and the predominant CD8 T-cell population was IFN-γ^+^IL-2^-^TNF-α^+^Perforin^-^ (Fig 3C).

In summary, these data indicate that ongoing helminth infection reduced the proportion of TB cases with detectable *Mtb*-specific CD8 T cells.

### Differences in systemic inflammation markers in patients from TZ and SA

**Fig 4:**
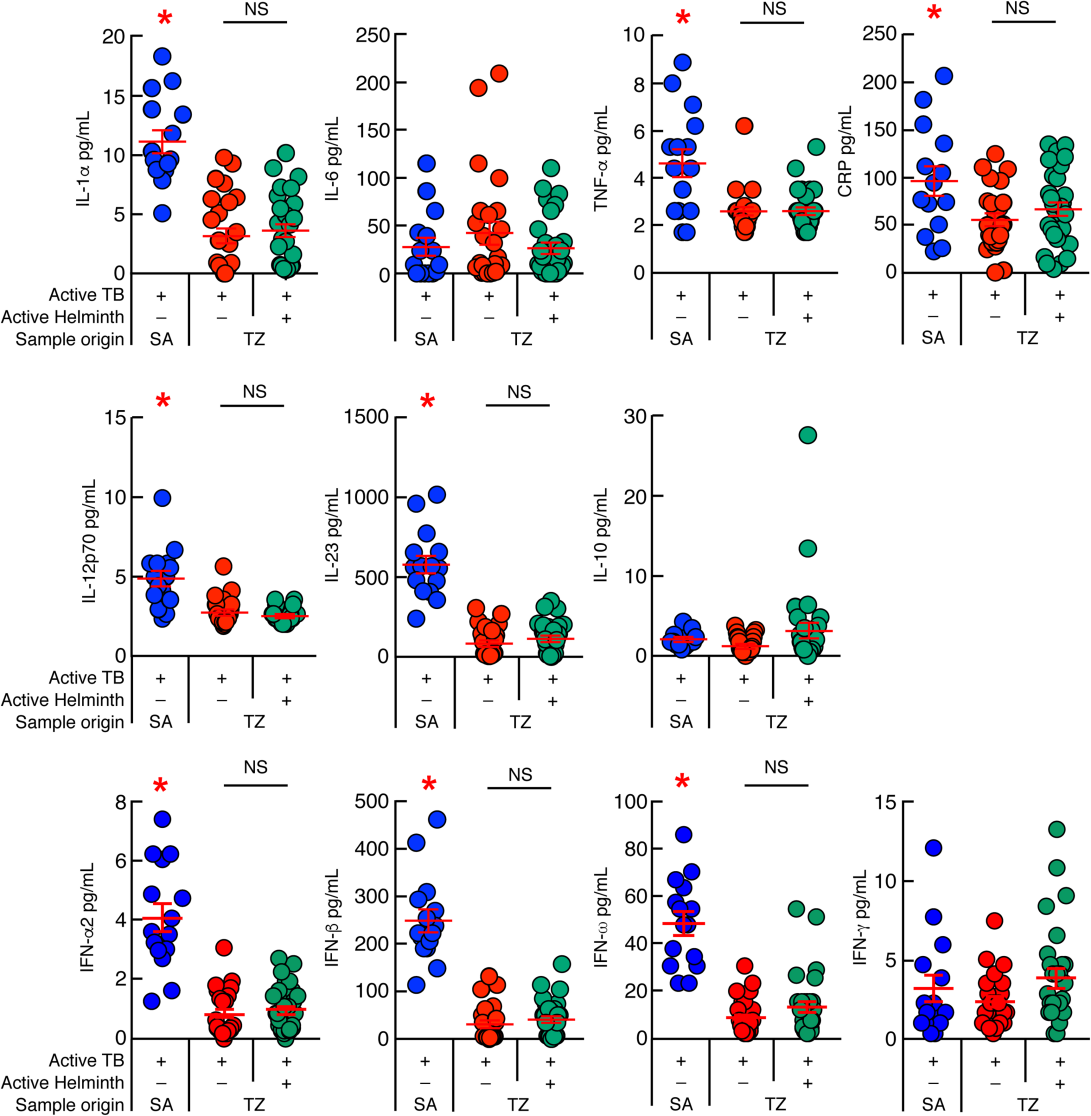
Differences in systemic inflammation markers in patients from TZ and SA. Serum levels of IL-1α, IL-6, TNF-α, CRP, IL-12p70, IL-23, IL-10, IFN-α2, IFN-β, IFN-ω and IFN-γ of TB patients from SA (n = 15), TB and *Mtb*/helminth co-infected patients from TZ (n = 25 and 30, respectively). TB patients were color coded; TB patients from SA, blue; TB patients from TZ, red and *Mtb*/helminth co-infected patients, green. Red stars indicate statistical significance. Statistical significance (* = *P*<0.05) was calculated using one way Anova Kruskal-Wallis test followed by a Mann–Whitney test.

One of the objectives of the present study was to determine whether ongoing helminth infection may influence the levels of systemic inflammation markers. In order to address this issue, we first assessed the serum levels of IL-1α, IL-6, TNF-α, IL-10, IL-12p70, IFN-α2, IFN-β, IFN-ω, IFN-γ, IL-23 and CRP of TB patients from TZ and SA by multiplex bead array analyses (Fig 4). The cumulative data indicated that the serum levels of IL-1α, TNF-α, IL-12p70, IFN-α2, IFN-β, IFN-ω, IL-23 and CRP were significantly increased in TB patients from SA as compared to TB patients from TZ (*P*<0.05) (Fig 4). However, the levels of IFN-γ, IL-6 and IL-10 were not statistically different between TB patients from SA and TZ (*P*>0.05; Fig 4). Interestingly, ongoing helminth infection did not further influence the serum levels of cytokines and CRP of TB patients from TZ (*P*>0.05) (Fig 4).

Taken together, these data suggest that TB patients from SA and TZ showed differences in serum cytokine profile. In comparison to Tanzanian TB cases, South African patients showed a more pronounced pro-inflammatory serum cytokine profile associated with high levels of type I IFNs.

### Th1 cytokine profiles are associated with elevated systemic inflammation markers

**Fig 5:**
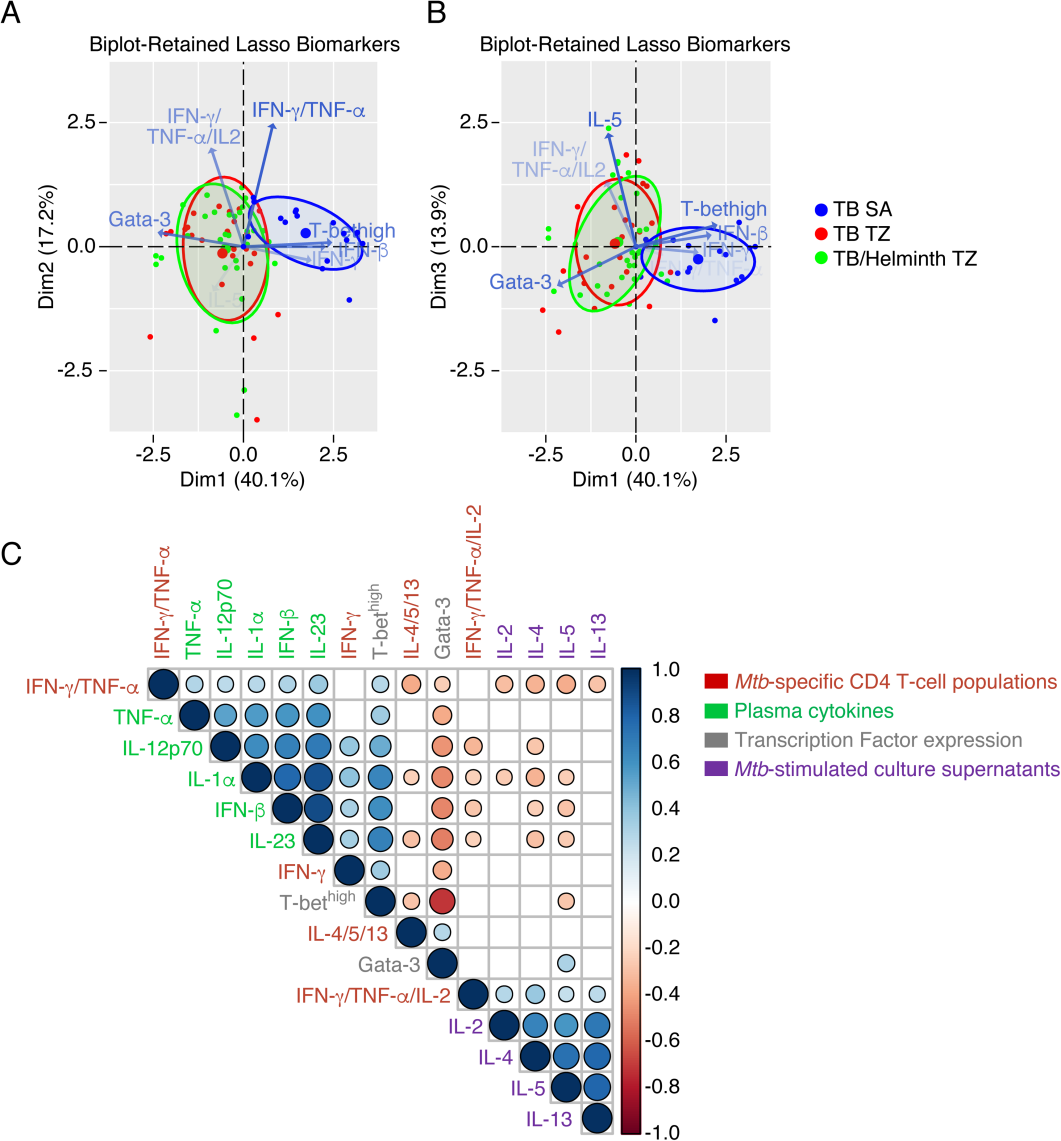
Th1 cytokine profiles are associated with elevated systemic inflammation markers. Principal component analysis (PCA) of *Mtb*-specific immunity determined in TB patients from SA (n = 15), TB and *Mtb*/helminth co-infected patients from TZ (n = 25 and 30, respectively). Blue, red and green symbols represent TB patients from SA, TB patients from TZ, and *Mtb*/helminth co-infected patients, respectively. Blue, red and green ellipses represent clusters formed by 70% of TB patients from SA, TB patients and *Mtb*/helminth co-infected patients from TZ, respectively. Blue arrows represent the differentially expressed immune parameters that significantly contributed (*P*<0.05) to discriminate *Mtb*-specific immune signatures (**A** and **B**). (**C**) Correlogram of imputed Spearman-Rank-Correlation between the proportion of triple IFN-γ/TNF-α/IL-2, dual IFN-γ/TNF-α, single IFN-γ and single IL-4/5/13 *Mtb*-specific CD4 T cells among total *Mtb*-specific CD4 T-cell responses, levels of IL-2, IL-4, IL-5 and IL-13 detected in *Mtb*-stimulated culture supernatants, percentages of memory CD4 T cells expressing Gata-3 or T-bet^high^, serum levels of IL-1α, TNF-α, IL-12p70, IFN-β and IL-23 determined in TB patients from SA and TZ (n = 70). Statistical significance was calculated using non parametric test. Empty squares correspond to non significant correlations (*P*>0.05), blue circles indicate direct correlations and red circles indicate inverse correlations (*P*<0.05). Blue and red circle sizes indicate statistical amplitude of correlation.

To better identify the immunological parameters associated with *Mtb*-specific immune signature in TB patients and the influence of ongoing helminth infection, a principal component analysis (PCA) was performed. The results indicated that the *Mtb*-specific immune profile of TB and *Mtb*/helminth co-infected patients from TZ clustered away from that of TB patients from SA with a percentage of discrimination reaching about 70% (Fig 5A and 5B). In depth analysis revealed that the differentially expressed immune parameters contributing the most to discriminate *Mtb*-specific immune response of TB patients from TZ and SA were the percentages of CD4 T cells expressing Gata-3 and T-bet^high^, the proportion of polyfunctional TNF-α/IFN-γ/IL-2, dual TNF-α/IFN-γ and single IFN-γ *Mtb*-specific CD4 T cells among total *Mtb*-specific CD4 T-cell responses, levels of IL-5 in *Mtb*-stimulated culture supernatants and IFN-β serum levels (Fig 5A and 5B and Table 2). Again, this analysis did not allow discriminating *Mtb*-specific immune response of TB and *Mtb*/helminth patients from TZ (Fig 5A and 5B).

**Table 2:** Contribution of the major differentially expressed immune parameters discriminating *Mtb*-specific immune signatures of TB patients from TZ and SA identified by principal component analysis.

Axes	Variance (%)	Immune parameters	Correlation	*P* value
Dim1	36.6%	T-bet^high^	0.827	3.4E-19
Dim1	36.6%	IFN-β	0.767	3.9E-15
Dim1	36.6%	IFN-γ	0.635	2.1E-09
Dim1	36.6%	IFN-γ/TNF-α	0.277	0.018
Dim1	36.6%	IL-5	-0.284	0.018
Dim1	36.6%	IFN-γ/TNF-α/IL2	-0.303	0.010
Dim1	36.6%	Gata-3	-0.800	3.5E-17
Dim2	18%	IFN-γ/TNF-α	0.835	7.5E-20
Dim2	18%	IFN-γ/TNF-α/IL2	0.670	1.2E-10
Dim2	18%	IL-5	-0.302	0.010
Dim3	15%	IL-5	0.838	4.2E-20
Dim3	15%	IFN-γ/TNF-α/IL2	0.483	1.7E-05
Dim3	15%	Gata-3	-0.288	0.014

Abbreviations: T-bet^high^, percentage of memory CD4 T cells expressing high level of T-bet; IFN-β, serum level of IFN-β; IFN-γ, proportion of single IFN-γ producing *Mtb*-specific CD4 T cells; IFN-γ/TNF-α, proportion of dual IFN-γ/TNF-α producing *Mtb*-specific CD4 T cells; IL-5, level of IL-5 detected in supernatants of *Mtb*-stimulated cell cultures; IFN-γ/TNF-α/IL-2, proportion of polyfunctional IFN-γ/TNF-α/IL-2 producing *Mtb*-specific CD4 T cells; Gata-3, percentage of memory CD4 T cells expressing Gata-3.

We next performed multiparametric statistical analysis to investigate the potential associations between the four major represented *Mtb*-specific CD4 T-cell populations *i*.*e*. triple IFN-γ/IL-2/TNF-α, dual IFN-γ/TNF-α, single IFN-γ and single IL-4/5/13 *Mtb*-specific CD4 T cells, the levels of cytokine detected in *Mtb*-stimulated culture supernatants, *i*.*e*. IL-2, IL-4, IL-5 and IL-13, the percentages of memory CD4 T cells expressing Gata-3 or T-bet^high^, and the serum levels of IL-1α, TNF-α, IL-12p70, IFN-β and IL-23 in TB patients from SA and TZ (Fig 5C). The combined data indicated that IFN-β serum concentrations positively correlated with i) T-bet^high^ memory CD4 T cells, ii) higher proportion of *Mtb*-specific Th1 cells (single IFN-γ and dual IFN-γ/TNF-α), iii) higher serum levels of pro-inflammatory cytokines (IL-1α and TNF-α), IL-12p70 and IL-23 and negatively correlated with a) Gata-3 expression on memory CD4 T cells, b) higher proportion of polyfunctional *Mtb*-specific CD4 T cells and c) type 2 cytokine secretion (IL-5 and IL-13) (*P*<0.05) (Fig 5C).

Taken together, the data indicate that the serum cytokine profile and the *Mtb*-specific immune signatures of TB patients from SA and TZ are significantly different and that Th1 cytokine profiles are positively associated with TB-induced systemic inflammation and higher serum levels of type I IFNs.

### Ongoing helminth infection is not associated with reduced anti-mycobacterial treatment outcome

**Fig 6:**
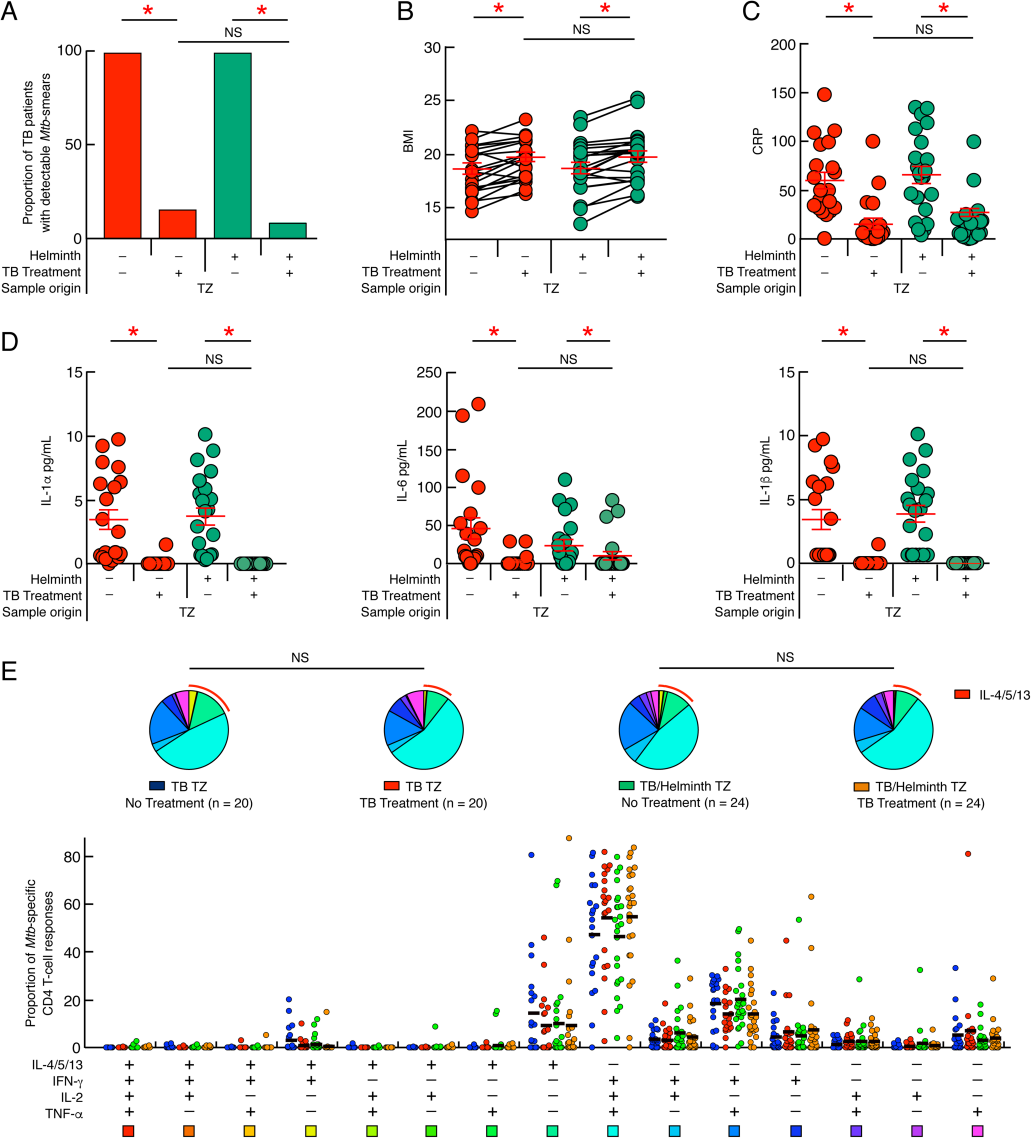
Influence of ongoing helminth infection on TB treatment efficiency. (**A**) Proportion of TB patients (n = 20) and *Mtb*/helminth (n = 24) co-infected patients with detectable *Mtb* prior to and following 60 days of TB treatment. Impact of TB treatment initiation on BMI (**B**), serum levels of CRP (**C**) and serum levels of IL-1α, IL-6 and IL-1β (**D**) of TB (n = 20) and *Mtb*/helminth (n = 22) co-infected patients from TZ prior to and following 60 days of TB treatment. Red and green circles represent TB and *Mtb*/helminth co-infected patients from TZ, respectively. (**E**) Functional profile of *Mtb*-specific CD4 T cells of TB patients (n = 20) and *Mtb*/helminth (n = 24) co-infected patients from TZ prior to and following 60 days of TB treatment. Blue, red, green and orange circles represent the proportion of *Mtb*-specific CD4 T cells producing TNF-α, IFN-γ, IL-2 and/or IL-4/5/13 in untreated TB patients, 60 days treated TB patients, untreated *Mtb*/helminth co-infected patients and 60 days treated *Mtb*/helminth co-infected patients recruited from TZ, respectively (**E**). TB patients were color coded; untreated TB patients, blue; treated TB patients, red; untreated *Mtb*/helminth co-infected patients, green; treated *Mtb*/helminth co-infected patients, orange. Red arcs identify IL-4/5/13 producing cell populations (**E**). Red stars indicate statistical significance (* = *P*<0.05). Statistical significance (*P*<0.05) was calculated using Chi square test (**A**), Wilcoxon signed-rank test (**B** and **C**) and one way Anova Kruskal-Wallis test followed by a Mann-Whitney test (**D**). Statistical analyses of the global cytokine profiles (pie charts, **E**) were performed by partial permutation tests using the SPICE software as described [97].

We then assessed the influence of ongoing helminth infection on TB drug treatment efficiency in the Tanzanian cohort. To address this issue, we first assessed the presence of *Mtb* in the sputum of treated individuals based on sputum smear microscopy. The cumulative data showed a significant reduction in the proportion of patients with detectable *Mtb* in the sputum following 60 days of drug treatment (*P*<0.05; Fig 6A). The presence of an ongoing helminth infection did not influence the effect of anti-mycobacterial drug treatment in relation to sputum detectable *Mtb* (*P*>0.05) (Fig 6A), suggesting that ongoing helminth infection was not associated with reduced anti-mycobacterial treatment efficiency.

In addition, 60 days of anti-mycobacterial treatment was associated with a significant increase of the body mass index (BMI) and significant decrease of serum levels of CRP and pro-inflammatory cytokines *i*.*e*. IL-1α, IL-6, and IL-1β (*P*<0.05; Fig 6B–6D), irrespective of the presence or absence of ongoing helminth infection (*P*>0.05) (Fig 6B–6D).

We next assessed the impact of TB treatment initiation on *Mtb*-specific T-cell immunity in TB and *Mtb*/helminth patients. The functional profiles of *Mtb*-specific CD4 T-cell responses and the transcription factor expression profiles were assessed using aforementioned experimental strategies. The cumulative data indicated that the initiation of efficient TB treatment did not significantly influence 1) the *Mtb*-specific CD4 T-cell functional profiles (*P>*0.05) (Fig 6E), 2) the nature and the amount of Th2 cytokines produced *i*.*e*. IL-4 and/or IL-5 and/or IL-13 (*P>*0.05) (S6A Fig) and 3) the transcription factor profile of TB and *Mtb*/helminth infected patients from TZ (*P>*0.05) (S6B Fig). Indeed, two months after TB treatment, the cytokine profile of *Mtb*-specific CD4 T cells was still dominated by polyfunctional IFN-γ/IL-2/TNF-α and Th2 *Mtb*-specific CD4 T cells (Fig 6E).

In summary, two months of TB treatment did not change the functional profile of *Mtb*-specific CD4 T cells. These data provide evidence that TB treatment outcome during this follow up period was not influenced by presence or absence of ongoing helminth infection.

## Discussion

Helminth infections have been shown to impact the control of virus replication in mouse models [52, 56] and interfere with vaccine induced immune responses [57, 58]. Since helminths and *Mtb* are co-endemic in several regions of the world, including Tanzania [1], we hypothesized that ongoing helminth infection may influence and potentially modulate the functional profile of *Mtb*-specific T-cell responses. Our hypothesis is founded on the observations that helminths stimulate Th2 type immune responses and previous studies that demonstrated that the cytokine micro-environment may influence, at least *in vitro*, the functional profile of antigen-specific CD4 T cell responses [59, 60].

In the present study, we provide evidence that a significant proportion of *Mtb*-specific CD4 T cells in patients with active TB disease from TZ have a Th2 cytokine profile as indicated by the production of IL-4/IL-5/IL-13 and expression of the Th2 cell lineage transcription factor Gata-3. *Mtb*-specific CD4 T-cells comprised Th1 and Th2 cells with a polyfunctional cytokine profile, and the increased frequency of Gata-3^+^ memory CD4 T cells was associated with reduced frequency of T-bet^high+^ memory CD4 T cells. Interestingly, *Mtb*-specific CD4 T cells in patients with active TB disease from SA had a typical Th1 profile encompassing single IFN-γ and dual IFN-γ/TNF-α cells. Taken together, these results demonstrate that active TB disease induced the generation of mixed Th1/Th2 *Mtb*-specific CD4 T cells in patients from TZ whereas a typical Th1 *Mtb*-specific CD4 T cell response was generated in patients from SA. Further investigations would be needed to determine whether TB-uninfected individuals from TZ and SA harbor different basic response to TB antigens.

These observations are of high interest since the assessment of *Mtb*-specific CD4 T-cell cytokine profile is consistently proposed to allow the discrimination between active and latent *Mtb* infections [61–64]. Among, these parameters, high proportions of IL-2-producing *Mtb*-specific CD4 T cells (in association with Th1 cytokines *i*.*e* IFN-γ and/or TNF-α) are associated with individuals with LTBI and therefore *Mtb* containment, while high proportion of *Mtb*-specific CD4 T cells producing Th1 cytokines in absence of IL-2 is associated with patients suffering from active TB disease [62–65]. Indeed, reduced capacity to produce IL-2 is usually associated with high antigen load, persistent T-cell stimulation, expression of co-inhibitory molecules and differentiation towards effector memory and/or terminally differentiated effector memory [66, 67]. Interestingly, CD4 T cells coexpressing IFN-γ and TNF-α and harboring a phenotype of effector-memory response were associated with active tuberculosis in HIV-uninfected [68, 69] and HIV-infected TB patients [67]. Of note, polyfunctional helminth-specific CD4 T-cell responses were also recently associated to helminth biological activity [47, 70].

As mentioned above, the study hypothesis was that helminth infection might influence the functional profile of *Mtb*-specific CD4 T cells. In this context, about 67% of patients from TZ either had an ongoing active helminth infection or had evidence of previous helminth exposure/infection. However, about 11% of the patients from SA also showed a positive serology for previous helminth exposure/infection with helminth but no sign of generation of *Mtb*-specific Th2 CD4 T cells. Despite this discordance between positive serology for helminth exposure and lack of the generation of *Mtb*-specific Th2 CD4 T cells in patients from SA, we cannot exclude that the frequency and/or severity of helminth re-infection/exposure in patients from TZ is higher as when compared to South African patients and therefore responsible for the mixed Th1/Th2 functional profile of *Mtb*-specific CD4 T cells [71, 72]. In addition, we cannot exclude that human or *Mtb* genetic diversity and other yet unknown environmental factors may contribute to the generation of mixed Th1/Th2 functional cytokine profile and/or influence the durability of the Th2 response after parasite infection is cleared [73] in patients from TZ.

Consistent with previous studies [53, 74], about 80% of TB patients from TZ had *Mtb*-specific CD8 T cell responses detected by polychromatic flow cytometry. Interestingly, this proportion was significantly reduced (about two fold) in the presence of ongoing helminth infections. These results provide additional evidence that helminth infections may significantly influence the generation of distinct T cell subsets in active TB disease. This important finding echoes with recent data demonstrating that ongoing helminth infection impairs virus-specific T-cell immunity *via* a STAT-6-dependent alternative activation of macrophages differentiation [52].

In contrast to Tanzanian TB cases, the South African TB patients were characterized by significantly higher serum levels of pro-inflammatory cytokines (IL-1α and TNF-α) in combination with IL-12p70 and type I IFNs. Interestingly, statistical analyses revealed that elevated serum levels of IFN-β were associated with elevated serum levels of pro-inflammatory cytokines and Th1 *Mtb*-specific CD4 T cells lacking IL-2 co-production. These IFN-β serum levels were inversely correlated with polyfunctional *Mtb*-specific CD4 T cells and Th2 cytokines detected by polychromatic flow cytometry and in cell culture supernatants. Based on these findings, the so far unappreciated role of IFN-β (and type I IFNs in general) in coordinating TB specific immunity needs to be further explored.

The role of Th1, Th2 and CD8 T cells in the control of *Mtb* infection and the progression of TB disease is under intense debate. It has been clearly demonstrated that functional impairment of the IL-12p70/IFN-γ axis predisposes to the development of mycobacterial disease [7–10], probably by compromising the phagocytic and cytolytic capacity of macrophages primed with IFN-γ [75]. The role of *Mtb*-specific CD8 T cells is however still controversial. Some studies indicate that *Mtb*-specific CD8 T cells may play an important role in protective immunity against TB *via* the production of perforin and/or cytolysin [76–80], while others indicate that the presence of *Mtb*-specific CD8 T cells may be detrimental [81], since *Mtb*-specific CD8 T cells were enriched in TB patients as compared to individuals with LTBI [53]. Excessive IFN-γ production by CD4 or CD8 T cells may in fact favour *Mtb* transmission *via* inflammation mediated mucosal damage enabling access of *Mtb* bacilli to airways [81].

The dual role of IFN-γ in the protection or progression of TB disease may be linked to macrophage hypo-responsiveness to IFN-γ [82], also called progressive exhaustion, which can be mediated by *Mtb*-induced type I IFNs [83, 84]. Indeed, recent studies demonstrated that *Mtb*-induced type I IFN might be detrimental, since TB patients with reduced/absent type I IFN signature had reduced bacterial load and/or improved host survival [85–87]. Interestingly, other studies indicate that type I IFN responses enhance CD4 T-cell differentiation towards Th1 [88], enhance CD8 T-cell responses [89] and interfere with IL-23-mediated Th17 cell differentiation and IL-4-mediated Th2-cell differentiation by inhibiting Gata-3 expression [88, 90, 91], supporting our observations.

The reason why TB patients from TZ had lower levels of type I IFNs remains unclear and needs to be further investigated. However, one could postulate that the genetic background of the *Mtb* strains isolated from TZ and SA, the antigen load and the genetic background of the individuals living in TZ *versus* SA might be associated with this profile, since the level of *Mtb*-induced type I IFN production might be strain dependent [92]. Of note, these parameters were not evaluated in the present study and would require further evaluation.

Finally, we did not observe any influence of ongoing helminth infection on the efficacy of TB therapy. Interestingly, after sixty days of treatment, we did not observe changes in *Mtb*-specific CD4 T-cell cytokine and transcription factor expression profile but strongly reduced CRP serum levels and pro-inflammatory cytokine circulation in combination with improved BMI.

In conclusion, we provide evidence that the generation of *Mtb*-specific CD4 and CD8 T cell responses, may be substantially influenced by co-infectious agents and possibly genetic and environmental factors resulting in pronounced variations in the qualitative and quantitative profile of pathogen-specific responding T cells in human populations.

## Materials and methods

### Study group, helminth diagnosis and cell isolation

In total, 72 subjects were recruited to participate in this study. No statistical method was used to predetermine sample size. Fifty-five subjects were recruited at the Mwananyamala Hospital, Dar es Salaam, and the TB clinic of Bagamoyo (TZ). TB patients (n = 55) were selected based on sputum smear microscopy confirmed by GeneXpert assay and HIV infection was ruled out by rapid serological test (Alere Determine HIV-1/2 test). The diagnosis of ongoing and/or past helminth infection was based on assays performed on feces (Kato-Katz thick smear, FLOTAC and Baermann assays), urine (urine filtration), whole blood (Immuno-chromatography) and serum (ELISA) samples at date of blood sample collection and the assay used depended on the helminth species and on the site of sample collection. All TB patients from TZ were screened for active soil-transmitted helminths (hookworms, *Ascaris lumbricoides*, *Trichuris trichiura*) and *S*. *mansoni* infections using the Kato-Katz thick smear and FLOTAC methods performed on one stool sample at date of blood sample collection, for active *S*. *stercoralis* infection using the Baermann technique and for active *S*. *haematobium* infection using the urine filtration method. Binax NOW ICT test card were used to detect *W*. *Bancrofti* antigen on blood sample [93, 94]. In addition, serology for 7 different helminths (*Echinococcus spp*, *Fasciola hepatica*, Filaria, *Schistosoma spp*, *S*. *stercoralis*, *Toxocara spp* and *Trichinella spp*) was performed by ELISA. The serodiagnostic helminth screening ELISA is routinely performed at the diagnostic centre of the Swiss Tropical and Public Health Institute and detects helminth specific IgG. A total of 30 *Mtb*/helminth co-infected patients were recruited. TB patient co-infected with only one helminth species were infected with *S*. *mansoni* (n = 7), *W*. *bancrofti* (n = 6), hookworms (n = 2), *S*. *haematobium* (n = 2) or *S*. *stercoralis* (n = 2). Eleven TB patients (36%) were co-infected with multiple helminth species. Blood samples were collected prior to and following 60 days of anti-mycobacterial treatment (“fixed dose combination” consisting of Rifampicin, Isoniazid, Pyrazinamide and Ethambutol, (RHZE)) from 20 TB and 24 *Mtb*/helminth co-infected patients recruited in TZ. In addition, seventeen subjects were enrolled at the field site of the South African Tuberculosis Vaccine Initiative in the Boland Overberg region of the Western Cape Province of SA (SA). TB disease was diagnosed by positive sputum Xpert MTB/RIF and HIV infection was ruled out by rapid serological test. PBMCs were collected as part of a cross-sectional study, in HIV-negative participants before commencing treatment for TB. Diagnosis of helminth exposure was performed using ELISA detecting helminth-specific IgG (*Echinococcus spp*, *Fasciola hepatica*, Filaria, *Schistosoma spp*, *S*. *stercoralis*, *Toxocara spp* and *Trichinella spp*).

### Ethics statement

All participants were adults and provided written informed consent and the study protocol was approved for TZ by the Ethikkomission beider Basel (EKBB; Basel, Switzerland; reference number 257/08), the Ifakara Health Institute Institutional Review Board and the National Institute for Medical Research (NIMR; Dar es Salaam, United Republic of Tanzania; reference number NIMR/HQ/R.8a/Vol.IX/1098). For SA, the Human Research Ethics Committee of the University of Cape Town granted the study protocol approval.

### Antibodies

The following monoclonal antibodies (mAbs) were used in different combinations. CD3-APC-H7 (CloneSK7), CD4-PECF594 or CD4-APC (Clone RPA-T4), CD8-PB (Clone RPA-T8), IFN-γ-AF700 or IFN-γ-APC (Clone B27), TNF-α-PeCy-7 (Clone MAb11), IL-4-PE (Clone 3010.211), IL-2-PE (Clone MQ1-17H12), Gata-3-PeCy-7 (Clone L50-823), all from Becton Dickinson (BD); CD45RA-BV711 (Clone HI100), IL-2-PerCpCy5.5 (Clone MQ1-17H12), IL-5-PE (Clone TRFK5), IL-13-PE (Clone JES10-5A2), T-bet-PerCpCy5.5 (Clone 4B10) were purchased from BioLegend; CD8-Efluor625NC (Clone RPA-T8) from eBioscience; perforin-FITC (Clone B-D48) from Diaclone.

### Antigens

*Mtb*-derived CFP-10 and ESAT-6 peptide pools are composed of 15-mers overlapping by 11 amino-acids encompassing the entire sequences of the proteins and all peptides were HPLC purified (>90% purity).

### *Ex vivo* assessment of CD4 T-cell cytokine profile by ICS

PBMCs were stimulated overnight in complete media (RPMI (Invitrogen), 10% fetal calf serum (FCS; Invitrogen), 100 µg/ml penicillin, 100 unit/ml streptomycin (BioConcept)) with ESAT-6 and CFP-10 peptide pools (1 µg/ml) or with *Staphyloccocus enterotoxin B* (SEB; 250 ng/mL) or unstimulated in the presence of Golgiplug (1 μl/ml; BD) as previously described [95]. At the end of the stimulation period, cells were washed and stained (20 min; 4°C) for dead cells using the Aqua LIVE/DEAD stain kit (Invitrogen), washed and stained (20 min; 4°C) with mAbs to CD3, CD4, CD8 and CD45RA. Cells were then permeabilized (30 min; 20°C) (Cytofix/Cytoperm, BD) and stained (20 min; 20°C) with mAbs to TNF-α, IFN-γ, IL-2, IL-4, IL-5 and IL-13.

### *Ex vivo* assessment of CD8 T-cell cytokine profile by ICS

PBMCs were stimulated overnight in complete media (RPMI (Invitrogen), 10% fetal calf serum (FCS; Invitrogen), 100 µg/ml penicillin, 100 unit/ml streptomycin (BioConcept)) with ESAT-6 and CFP-10 peptide pools (1 µg/ml) or *Staphyloccocus enterotoxin B* (SEB; 250 ng/mL) or unstimulated in the presence of Golgiplug (1 μl/ml; BD). At the end of the stimulation period, cells were washed and stained (20 min; 4°C) for dead cells using the Aqua LIVE/DEAD stain kit, then permeabilized (30 min; 20°C) (Cytofix/Cytoperm, BD) and stained (20 min; 20°C) with mAbs to CD3, CD4, CD8, TNF-α, IFN-γ, IL-2 and perforin.

### Assessment of T-bet, Gata-3 expression

PBMCs were washed, stained (20 min; 4°C) for dead cells using the Aqua LIVE/DEAD stain kit, then washed and stained (20 min; 4°C) for CD3, CD4, CD8, CD45RA. Cells were then washed, permeabilized (45 min; 4°C) (Foxp3 Fixation/Permeabilization Kit; eBioscience) and stained (20 min; 4°C) with mAbs to T-bet and Gata-3.

### Assessment of *Mtb*-stimulated culture supernatant cytokine profile by luminex assay

PBMCs (2x10^5^ cells) were stimulated for 24 hours in complete media (RPMI (Invitrogen), 10% fetal calf serum (FCS; Invitrogen), 100 µg/ml penicillin, 100 unit/ml streptomycin (BioConcept)) with ESAT-6 and CFP-10 peptide pools (1 µg/ml) or with *Staphyloccocus enterotoxin B* (SEB; 250 ng/mL) or left unstimulated (negative control). At the end of the stimulation period, culture supernatants were collected and levels of TNF-α, IFN-γ, IL-2, IL-4, IL-5, IL-13, IL-10, IL-17A and IL-17F were assessed cells by luminex assay (ProcartaPlex Mix&Match Human plex, eBioscience).

### Assessment of serum cytokine profile

Serum levels of IL-1α, IL-6, TNF-α, IL-12p70, IL-23, IL-10, IFN-α2, IFN-β, IFN-ω and IFN-γ was assessed by luminex assay (ProcartaPlex Mix&Match Human plex, eBioscience) and CRP was assessed by nephelemetry (CardioPhasehsCRP, Siemens Healthcare Diagnostics Products GmbH) as previously described [96].

### Flow cytometry analyses

Cells were fixed with CellFix (BD), acquired on an LSRII SORP (4 lasers: 405, 488, 532 and 633 nm) and analyzed using FlowJo (version 9.7.7) (Tree star Inc, Ashland, OR, USA). Frequencies of cytokine-producing *Mtb*-specific T cells and cytokine profile of *Mtb*-specific T-cell responses were analyzed using SPICE software (version 5.34) following background subtraction. When required, analysis and presentation of distributions was performed using SPICE, downloaded from <http://exon.niaid.nih.gov/spice> [97].

### Statistical analyses

Statistical significance (*P* values) was obtained either using two-tailed Chi-square analysis for comparison of positive proportions or using one-way ANOVA (Kruskal-Wallis test) followed by Mann–Whitney test or Wilcoxon Matched-pairs two-tailed Signed Rank test for multiple comparisons or Spearman rank test for correlations using GraphPad Prism version 7 (San Diego, CA). Statistical analyses of global cytokine profiles (pie charts) were performed by partial permutation tests using the SPICE software as described [97]. Principal component analysis (PCA) was performed using the R ‘‘stats” package. To normalize data distribution, the values of each parameter were first log transformed. Data were then filtered using Lasso method [98].

## Supporting information

S1 Fig Flow chart of the enrolled patients. (PDF)

S2 Fig Gating strategy used to assess CD4 T-cell cytokine profile. (PDF)

S3 Fig *S*. *mansoni* soluble egg antigen-specific CD4 T cells from TB/*S*. *mansoni* co-infected patients from TZ. Proportion of SEA-specific CD4 T-cell responses producing IFN-γ, IL-4/5/13, TNF-α and/or IL-2 of TB patients from TZ (n = 7). All the possible combinations of the responses are shown on the x axis and the percentage of the functionally distinct cell populations within the SEA-specific CD4 T-cell populations are shown on the y axis. Responses are grouped and color-coded on the basis of the number of functions. The pie chart summarizes the data, and each slice corresponds to the fraction of SEA-specific CD4 T cell response with a given number of functions within the responding CD4 T-cell population. Bars correspond to the fractions of different functionally distinct CD4 T-cell populations within the total CD4 T cells. Red arcs correspond to IL-4/5/13-producing CD4 T-cell populations.
(PDF)

S4 Fig Influence of the helminth species, polyparasitism, helminth species harbouring lung migration capacity and past helminth exposure on the proportion of *Mtb*-specific CD4 T cells producing Th2 cytokines, on the level of Th2 cytokines secreted and on the percentage of CD4 T cells expressing Gata-3. (**A**) Proportion of *Mtb*-specific CD4 T cells producing IL-4/5/13 among total *Mtb*-specific CD4 T-cell responses assessed in TB patients from TZ infected with one helminth (n = 19) as compared to TB patients from TZ infected with more than one helminth (n = 11) or (**B**) between TB patients from TZ infected with helminth species harbouring (hookworm and *S*. *stercoralis*) or not (*S*. *mansoni*, *S*. *haematobium* and *W*. *bancrofti*) lung migration capacity. (**C**) Proportion of *Mtb*-specific CD4 T cells producing IL-4/5/13 among total *Mtb*-specific CD4 T-cell responses assessed in TB patients from SA with (n = 2) or without past helminth exposure (n = 15). (**D**) Proportion of *Mtb*-specific CD4 T cells producing IL-4/5/13 among total *Mtb*-specific CD4 T-cell responses assessed in TB patients from TZ without ongoing helminth infection but with (n = 7) or without past helminth exposure (n = 18). (**E**) Levels of IL-4, IL-5 and IL-13 secreted in *Mtb*-stimulated culture supernatants in TB patients from TZ infected with one helminth (n = 19) and TB patients from TZ infected with more than one helminth (n = 10) or (**F**) between TB patients from TZ infected with helminth species harbouring or not lung migration capacity. (**G**) Levels of IL-4, IL-5 and IL-13 secreted in *Mtb*-stimulated culture supernatants in TB patients from SA with (n = 2) or without past helminth exposure (n = 10). (**H**) Levels of IL-4, IL-5 and IL-13 secreted in *Mtb*-stimulated culture supernatants in TB patients from TZ without ongoing helminth infection but with (n = 6) or without past helminth exposure (n = 15). (**I**) Percentage of memory CD4 T cells (CD45RA^-^) expressing Gata-3 of TB patients from TZ infected with one helminth (n = 8) and TB patients from TZ infected with more than one helminth (n = 5) or (**J**) between TB patients from TZ infected with helminth species harbouring or not lung migration capacity. (**K**) Percentage of memory CD4 T cells (CD45RA^-^) expressing Gata-3 of TB patients from SA with (n = 2) or without past helminth exposure (n = 10). (**L**) Percentage of memory CD4 T cells (CD45RA^-^) expressing Gata-3 of TB patients from TZ without ongoing helminth infection but with (n = 6) or without past helminth exposure (n = 8). Helminth species were color coded (**A**, **E** and **I**); *W*. *bancrofti*, orange; hookworms, green; *S*. *mansoni*, red, *S*. *haematobium*, yellow, *S*. s*tercolaris*, violet, and patients infected with more than one helminth species in light blue. Statistical significance (*P*<0.05) was calculated by Mann-Whitney test.
(PDF)

S5 Fig The percentage of memory CD4 T cells expressing T-bet^high^ inversely correlates with the percentage of memory CD4 T cells expressing Gata-3. (A) Correlation between the percentage of memory CD4 T cell expressing Tbet^high^ and the percentage of memory CD4 T cell expressing Gata-3 in TB patients from SA (n = 12), TB patient (n = 14) and *Mtb*/helminth co-infected patients (n = 13) from TZ. TB patients were color coded; TB patients from SA, blue; TB patients from TZ, red and *Mtb*/helminth co-infected patients, green. Statistical significance (*P*<0.05) was calculated using Spearman rank test.
(PDF)

S6 Fig Impact of anti-mycobacterial treatment on the Th2 cytokine secretion and on transcription factor expression. (**A**) Levels of IL-4, IL-5 and IL-13 produced in *Mtb*-stimulated culture supernatants of TB (n = 13) and *Mtb*/helminth co-infected patients from TZ (n = 18) assessed by luminex assay. (**B**) Percentage of memory (CD45RA^-^) CD4 T cells expressing Gata-3 or T-bet^high^ of TB (n = 11) and TB/helminth co-infected patients (n = 8) from TZ. TB patients were color coded; TB patients, red and *Mtb*/helminth co-infected patients, green.
(PDF)
