# Supplementary figures and images for: Mixed Th1 and Th2 Mycobacterium tuberculosis-specific CD4 T cell responses in patients with active pulmonary tuberculosis from Tanzania
Source: PLoS Negl Trop Dis. 2017 Jul 31;11(7):e0005817. doi: 10.1371/journal.pntd.0005817 (PMC5552332; doi:10.1371/journal.pntd.0005817)

## Supplemental Figure 1

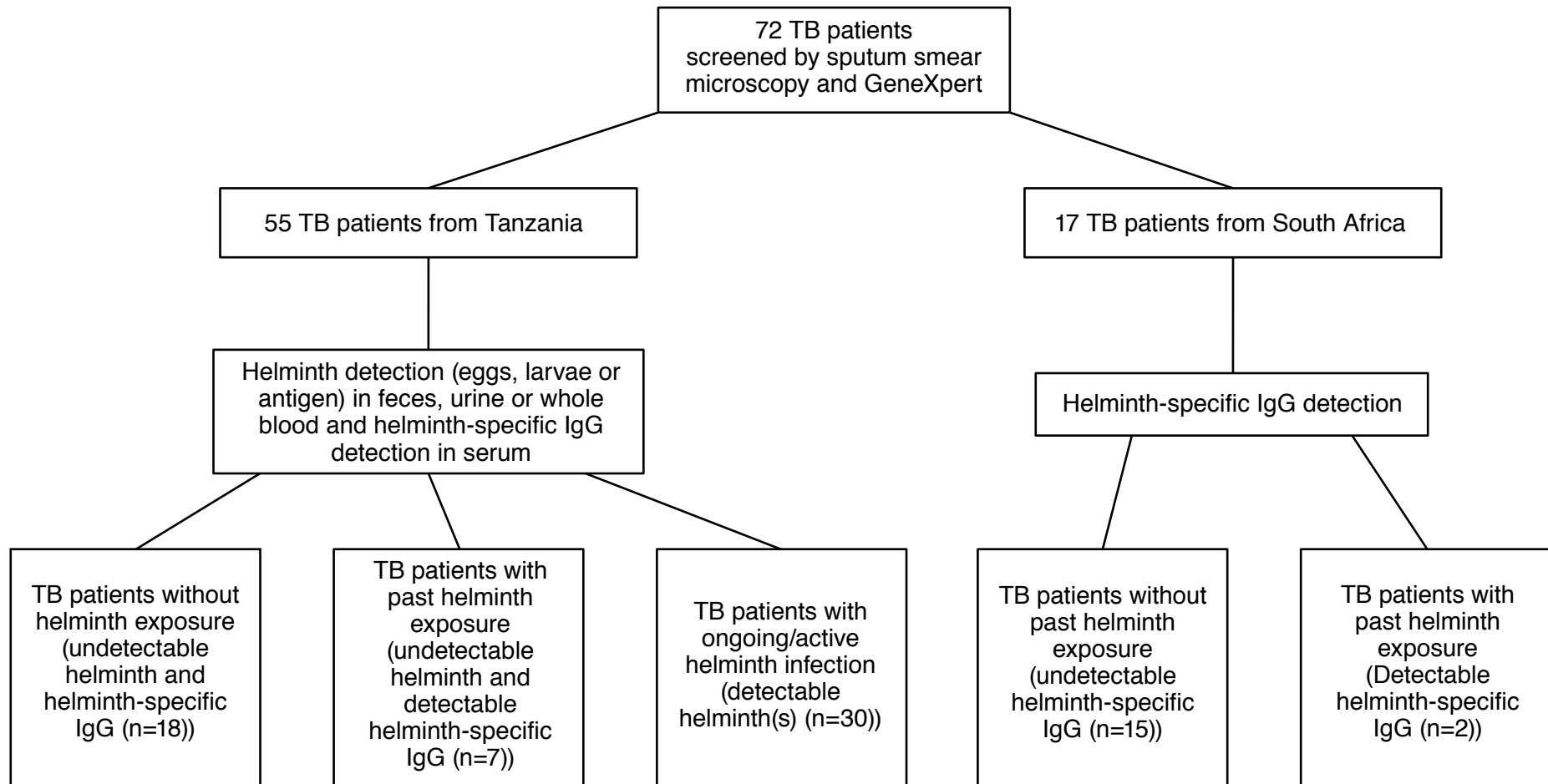

Supplement: S1 Fig — (PDF) [file pntd.0005817.s001.pdf]

# Supplemental Figure 2

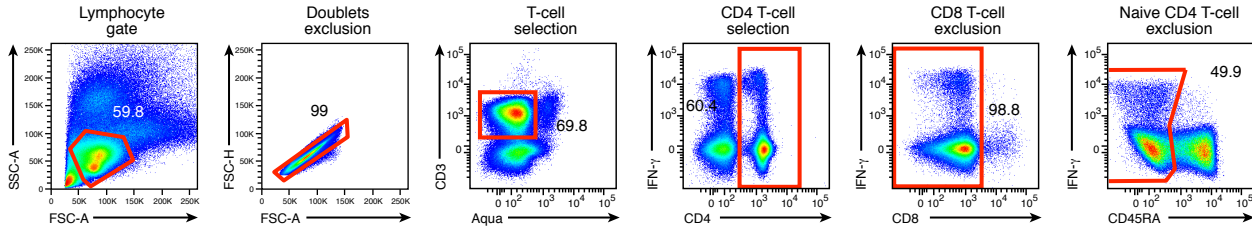

Supplement: S2 Fig — (PDF) [file pntd.0005817.s002.pdf]

# Supplemental Figure 3

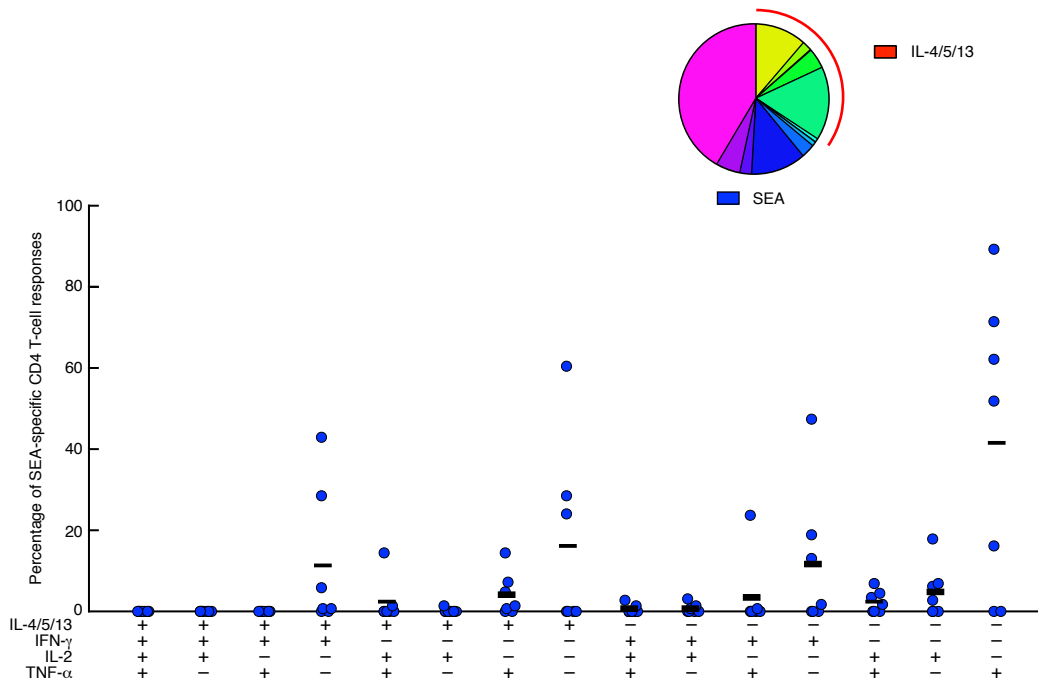

Supplement: S3 Fig — Proportion of SEA-specific CD4 T-cell responses producing IFN-γ, IL-4/5/13, TNF-α and/or IL-2 of TB patients from TZ (n = 7). All the possible combinations of the responses are shown on the x axis and the percentage of the functionally distinct cell populations within the SEA-specific CD4 T-cell populations are shown on the y axis. Responses are grouped and color-coded on the basis of the number of functions. The pie chart summarizes the data, and each slice corresponds to the fraction of SEA-specific CD4 T cell response with a given number of functions within the responding CD4 T-cell population. Bars correspond to the fractions of different functionally distinct CD4 T-cell populations within the total CD4 T cells. Red arcs correspond to IL-4/5/13-producing CD4 T-cell populations. (PDF) [file pntd.0005817.s003.pdf]

# Supplemental Figure 4

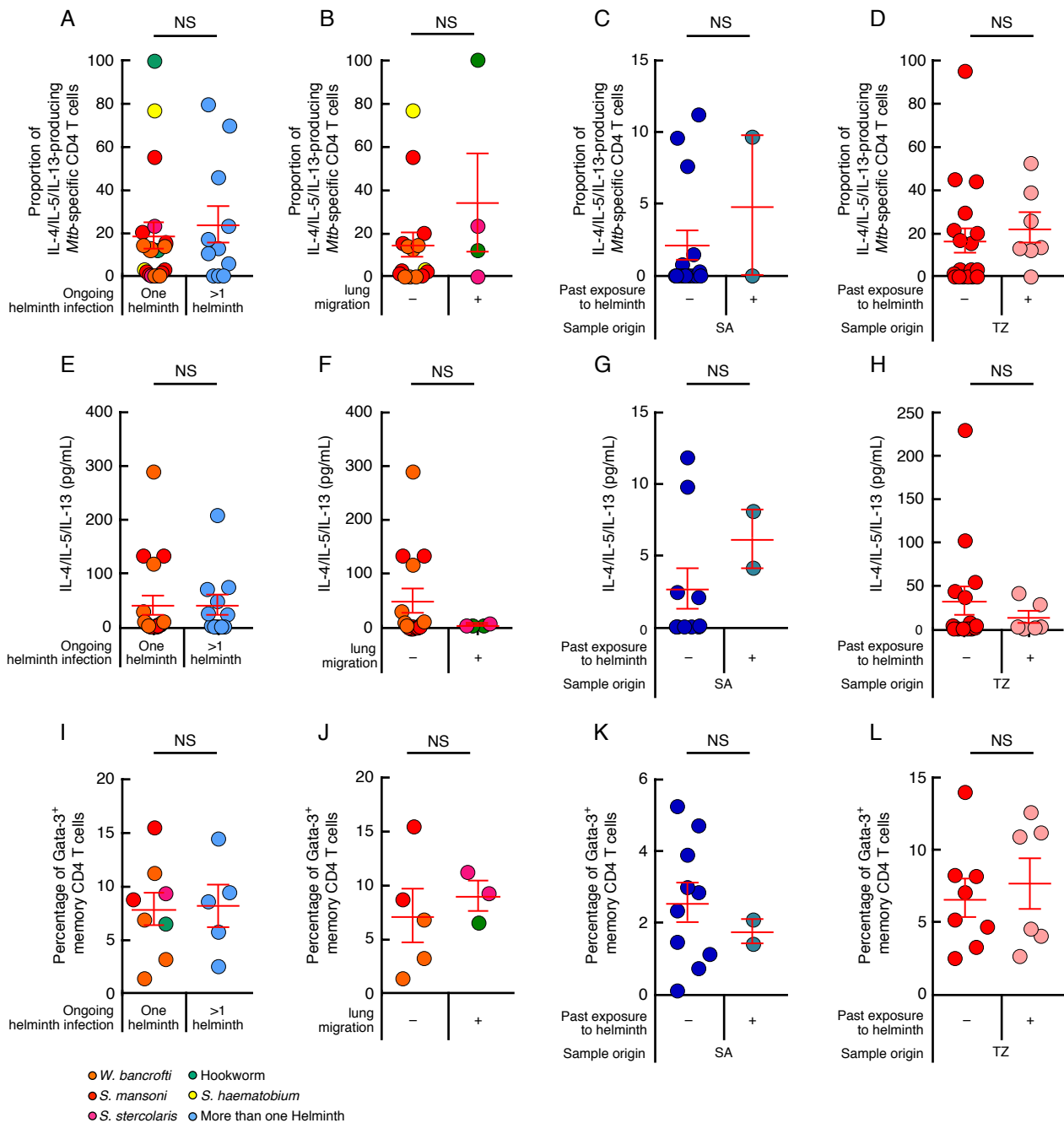

Supplement: S4 Fig — (A) Proportion of Mtb-specific CD4 T cells producing IL-4/5/13 among total Mtb-specific CD4 T-cell responses assessed in TB patients from TZ infected with one helminth (n = 19) as compared to TB patients from TZ infected with more than one helminth (n = 11) or (B) between TB patients from TZ infected with helminth species harbouring (hookworm and S. stercoralis) or not (S. mansoni, S. haematobium and W. bancrofti) lung migration capacity. (C) Proportion of Mtb-specific CD4 T cells producing IL-4/5/13 among total Mtb-specific CD4 T-cell responses assessed in TB patients from SA with (n = 2) or without past helminth exposure (n = 15). (D) Proportion of Mtb-specific CD4 T cells producing IL-4/5/13 among total Mtb-specific CD4 T-cell responses assessed in TB patients from TZ without ongoing helminth infection but with (n = 7) or without past helminth exposure (n = 18). (E) Levels of IL-4, IL-5 and IL-13 secreted in Mtb-stimulated culture supernatants in TB patients from TZ infected with one helminth (n = 19) and TB patients from TZ infected with more than one helminth (n = 10) or (F) between TB patients from TZ infected with helminth species harbouring or not lung migration capacity. (G) Levels of IL-4, IL-5 and IL-13 secreted in Mtb-stimulated culture supernatants in TB patients from SA with (n = 2) or without past helminth exposure (n = 10). (H) Levels of IL-4, IL-5 and IL-13 secreted in Mtb-stimulated culture supernatants in TB patients from TZ without ongoing helminth infection but with (n = 6) or without past helminth exposure (n = 15). (I) Percentage of memory CD4 T cells (CD45RA-) expressing Gata-3 of TB patients from TZ infected with one helminth (n = 8) and TB patients from TZ infected with more than one helminth (n = 5) or (J) between TB patients from TZ infected with helminth species harbouring or not lung migration capacity. (K) Percentage of memory CD4 T cells (CD45RA-) expressing Gata-3 of TB patients from SA with (n = 2) or without past helminth exposure [file pntd.0005817.s004.pdf]

# Supplemental Figure 5

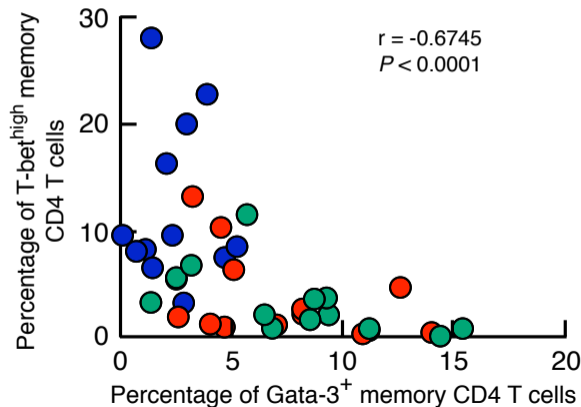

Supplement: S5 Fig — (A) Correlation between the percentage of memory CD4 T cell expressing Tbethigh and the percentage of memory CD4 T cell expressing Gata-3 in TB patients from SA (n = 12), TB patient (n = 14) and Mtb/helminth co-infected patients (n = 13) from TZ. TB patients were color coded; TB patients from SA, blue; TB patients from TZ, red and Mtb/helminth co-infected patients, green. Statistical significance (P<0.05) was calculated using Spearman rank test. (PDF) [file pntd.0005817.s005.pdf]

# Supplemental Figure 6

A

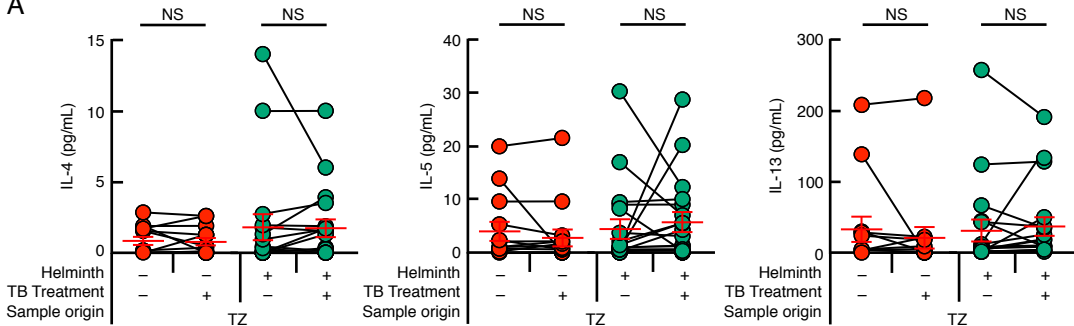

B

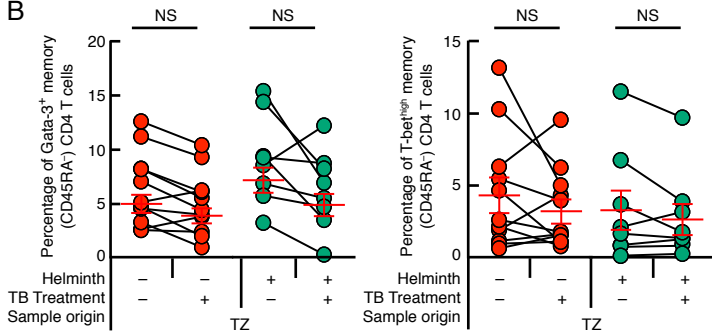

Supplement: S6 Fig — (A) Levels of IL-4, IL-5 and IL-13 produced in Mtb-stimulated culture supernatants of TB (n = 13) and Mtb/helminth co-infected patients from TZ (n = 18) assessed by luminex assay. (B) Percentage of memory (CD45RA-) CD4 T cells expressing Gata-3 or T-bethigh of TB (n = 11) and TB/helminth co-infected patients (n = 8) from TZ. TB patients were color coded; TB patients, red and Mtb/helminth co-infected patients, green. (PDF) [file pntd.0005817.s006.pdf]
